# The Molecular Data Organization for Publication (MDOP) R package to aid the upload of data to shared databases

**DOI:** 10.3897/BDJ.8.e50630

**Published:** 2020-04-23

**Authors:** Robert G Young, Jiaojia Yu, Marie-José Cote, Robert H Hanner

**Affiliations:** 1 University of Guelph, Guelph, Canada University of Guelph Guelph Canada; 2 Canadian Food Inspection Agency, Ottawa, Canada Canadian Food Inspection Agency Ottawa Canada

**Keywords:** Molecular database, DNA barcode, molecular sequence data, data organization tools, BOLD

## Abstract

Molecular identification methods, such as DNA barcoding, rely on centralized databases populated with morphologically identified individuals and their referential nucleotide sequence records. As molecular identification approaches have expanded in use to fields such as food fraud, environmental surveys, and border surveillance, there is a need for diverse international data sets. Although central data repositories, like the Barcode of Life Datasystems (BOLD), provided workarounds for formatting data for upload, these workarounds can be taxing on researchers with few resources and limited funding. To address these concerns, we present the Molecular Data Organization for Publication (MDOP) R package to assist researchers in uploading data to public databases. To illustrate the use of these scripts, we use the BOLD system as an example. The main intent of this writing is to assist in the movement of data, from academic, governmental, and other institutional computer systems, to public locations. The movement of these data can then better contribute to the global DNA barcoding initiative and other global molecular data efforts.

## Introduction

The use of molecular identification techniques on biological samples has been in practice for some time and includes methods like restriction fragment length polymorphisms (Magee et al. 1987), denaturing gradient gel electrophoresis (Muyzer et al. 1995), temperature gradient gel electrophoresis (Ogier et al. 2002), and analytical methods using molecular DNA sequence data (Bartlett and Davidson 1992). Generally, these methods use molecular data to ascertain the species or higher taxonomic identity of specimens through comparison to an established data set of known records. Technological advancements in DNA sequencing have reduced the cost, and by extension, increased the accessibility of obtaining nucleotide sequences from biological samples. Such advancements have led to an increase in the production of molecular sequence data resulting in the establishment and maintenance of large data repositories to house and share these data.

There are numerous large molecular databases including the National Center for Biotechnology Information (NCBI) GenBank, European Molecular Biology Laboratory (EMBL) Nucleotide Sequence Database, DNA Data Bank of Japan (DDBJ), and Barcode of Life Datasystems (BOLD). To contribute to these repositories users must follow specific upload guidelines and data formats. Following these guidelines can be challenging to end users. The scope of these challenges is often exacerbated when collaborating with researchers on large data projects over long periods of time. The inclusion of metadata can make adding records to these databases even more demanding. Metadata is data associated with primary sequence data, such as GPS location of collection, source of the DNA (such as tissue type), image files of the specimen, chromatograph files, and a multitude of other possibilities.

One of the data systems with more complex uploading processes, largely due to the rich metadata and files associated with records, is BOLD (Ratnasingham and Hebert 2007). The BOLD system houses DNA barcodes, which are small fragments of one or a few molecular DNA regions common across a taxonomic group or groups that are used to identify a specimen to a species (Hebert et al. 2003a, Hebert et al. 2003b). A DNA barcoding approach relies on centralized databases with geographically dispersed collections to account for as many possible variants of DNA present within each species. Only with diverse geographically-spread data do we begin to understand the barcode sequence variation within species compared to the variation between species, termed the barcode gap (Meyer and Paulay 2005). BOLD has records for a large diversity of multicellular organisms, linking a global community of researchers. These records include elements such as barcode sequences, taxonomic identifications, sequence chromatograms, specimen images, the location of publicly accessible reference specimens or tissues, among other metadata. This integration of data elements into molecular sequence databases is exceedingly important. The increased availability of such information can corroborate morphological identifications and can be valuable for future research.

Owing to its accuracy, universal nature, and more standardized methodology as compared to traditional morphological identifications, DNA barcoding has been successfully applied to a wide range of fields of study including taxonomy (Hubert and Hanner 2015), evolution (Mitterboeck et al. 2016), food mislabeling (Wong and Hanner 2008), food fraud (Naaum et al. 2018), biodiversity (Miller et al. 2016), ecology (Valentini et al. 2009), and border surveillance (Madden et al. 2019). As the methodology increases in use across diverse fields, so too does the necessity to build sequence databases with quality records and well populated metadata. Sustained interest in these research areas along with the contribution of associated metadata has the potential to deepen our understanding of aspects of biology, such as the presence of cryptic species, novel sequences representing undescribed species, and the use of synonymous names, the results of which can clarify our understanding of global biodiversity (DeSalle and Goldstein 2019).

In addition, the adoption of DNA barcoding for regulatory applications has placed barcoding in the context of state and international laws (Bonants et al. 2010, Thomas et al. 2016, Yancy et al. 2008). Governmental agencies have begun to adopt DNA barcoding to assist identifications, including the USA Food and Drug Administration and their efforts to identify seafood (Handy et al. 2011). The European Union’s Plant Protection Organization (EPPO) and the QBOL project have one of the more advanced programs using DNA barcoding standards and resources. The QBOL project supports the use of DNA barcoding for the identification of plant pathogenic quarantine organisms and Q-Bank was established as the central database resource for curated DNA barcodes used by QBOL (Bonants et al. 2010).

Populating public databases remains the main challenge for a growing community of researchers, regulators, and others using molecular identifications in a DNA barcoding approach. Scientists, who intend to share their data publicly, need to invest time to organize their data files to fulfill upload requirements. Although this can sometimes be labour-intensive, the ongoing management of this data in a centralized location provides great value when research teams shift or change, when data sharing across distances, and when publishing final data sets to accompany peer-reviewed literature. Although the strengths of a centralized DNA barcoding initiative are clear, there are still relatively few researchers contributing to public databases; and even when researchers contribute, they have been slow to make these data accessible to all users (Madden et al. 2019).

Although the DNA barcoding community has addressed potential roadblocks to the movement of data to shared databases through upload processes, challenges remain. This is especially true of researchers new to DNA barcoding or with few skills or resources to informatically bring data together in a standardized manner. For these researchers, hiring bioinformaticians can be expensive. In addition, some available options to organize data, such as using commands via a terminal window or command prompt (i.e. DOS C:\) may not always be possible when working in government or industry, where cybersecurity measures limit access (Barcode of Life Datasystems 2019). The R programming language is ideally suited to address the need for accessible and simple tools to help organize data for upload. R is a freely available open source program widely used to assist in the organization of data for various biological fields.

There are multiple R packages developed for manipulating genomic data (Foster et al. 2017, Pagès et al. 2019, Paradis and Schliep 2018) and others with specific foci on DNA barcoding and BOLD (Brown et al. 2012, Chamberlain 2019, Zhang et al. 2016). However, these packages and programs largely focus on post-upload analyses or downloading data already present on shared databases, as opposed to providing upload assistance. In addition, many R packages require knowledge of R, package functions, and file data formats to implement, which may be beyond some users’ abilities. The expertise required to navigate these more complex tools may be limiting the inclusion of records from researchers with limited experience in using such tools and limited time to learn complex options. This is especially concerning for taxonomists whose identified specimen records are essential to building high quality DNA barcode libraries.

To address these gaps, we present the package Molecular Data Organization for Publication (MDOP) (R Ver. 3.5.1; R Core Team 2018) to assist users in the standardization and upload of molecular sequence data and associated metadata files to centralized data repositories. The functions in MDOP were designed and tested on Windows 10 and tested on Mac OS Mojave Version 10.14.6. MDOP functions can help facilitate the organization of files (specimen images or trace files) and the pre-processing of DNA sequence files. This package contains functions which require little knowledge of R beyond calling one of the nine functions and reading directions. This package, installation instructions, and all of its associated functions are available on Github (https://github.com/rgyoung6/MDOP). In this article, we describe the purpose of each function and how they can help in the DNA barcode data submission process.

## Upload tools

The following three sections describe scenarios where data or files need to be manipulated or obtained. The functions in this package can be initiated through use of one or more arguments when initiating the function. However, if so desired the user does have the option to initiate the function without arguments and will be prompted for the necessary information (see the package-associated readme file for examples https://github.com/rgyoung6/MDOP/blob/master/README.md).

### Obtaining file lists

When preparing uploads to centralized databases, a list of all files is often needed with associated information (e.g., image file data, trace file data). These data can be obtained using DOS or IOS commands. However, the use of the command prompt is not always possible, particularly in places where security features limit this possibility such as government institutions and industry.

target_file_list()

This function lists files with the extensions JPG, AB1, or FASTA/FAS for a chosen directory and all subdirectories. The list of file paths and file names will be saved as a text file in the chosen directory. The user can either choose to submit the file path and the file type as arguments when initiating the tool or, alternatively, can run the tool with no arguments and be prompted for the necessary information. If running without arguments, the user will first be prompted to choose a file folder as a location to save the output file. Then it is necessary to input the type of file for which you would like to have a list (JPG, AB1, or FAS). The output for the script will appear in a text file with the naming convention YYYYMMDD_target_file_list_TYP.txt, where the first eight characters represent the date of running, the second section is in reference to the function name, and the final section with TYP is the file type chosen (JPG, AB1, or FAS).

### File Manipulation

Moving, copying, sorting, and subsetting large numbers of files is often necessary when preparing to upload data to shared databases. The organization of files for upload is made more difficult when processing files from multiple sources, research groups, and over time. These three functions can assist in the organization of diverse sets of files for upload.

recursive_copy()

Often, the submission of numerous files, including image and chromatogram (trace) files, to a centralized data system is necessary. Bringing files into a central folder may be difficult when dealing with large numbers of files stored in cascading file structures. recursive_copy() is written to bring all files with a specific extension into a central location thereby making it easier to upload these files. The recursive_copy() function copies files with the extensions JPG, AB1, or FASTA/FAS in a directory and all subdirectories and places these files in a single destination folder. The user can either choose to submit the file path and the file type as arguments when initiating the tool or can run the tool with no arguments and be prompted for the necessary information. If running without arguments, the user will first be prompted to choose a file folder where the new folder of copied files will be located. Then it is necessary to input the type of file for which you would like to copy the files (JPG, AB1, or FAS). The output for the script will appear in a file folder with the naming convention YYYYMMDD_ recursive_copy()_TYP, where the first eight characters represent the date of running, the second section is in reference to the function name, and the final section with TYP is the file type chosen (JPG, AB1, or FAS).

*max_packs()*

Uploads of image files to centralized databases are often limited to a particular size per upload. It can be time consuming and challenging to partition files into folders of target sizes. The max_packs() function can be utilized to create these partitioned folders quickly and easily. This function will take a single file folder (but not containing folders) with target files (JPG, AB1, or FAS) and distribute them into folders based on a maximum folder size. The user can either choose to submit the file path, file type, and maximum desired file folder size as arguments when initiating the tool or can run the tool with no arguments and be prompted for the necessary information. If running without arguments, the user will first be prompted to choose a file folder with the target files of interest. Then it is necessary to input the type of file for which you would like to copy the files (JPG, AB1, or FAS). Finally, the user will be required to input an integer value for the maximum allowable size for the folders created with the copied files. The outputs for the script will appear in the target file folder location with the naming convention YYYYMMDD_ max_packs_TYP_#, where the first eight characters represent the date of running, the second section is in reference to the function name, the third element TYP is the file type chosen (JPG, AB1, or FAS), and the final element # is an index for the folder number.

*copy_by_list()*

It is likely, after scrutiny, that some files associated with molecular records will not need to be uploaded to shared databases due to quality filtering. For example, if a DNA sequence was of poor quality it might be removed from the dataset for potential upload. This would then require the removal of associated metadata files. It is often time consuming to complete a point-and-click removal for all these records. In addition, the screening of these poor-quality records is often completed in fasta files and/or through the use of lists. copy_by_list() will assist in the copying of select files in a larger file folder and placing them into a new file folder based on a specified list. This tool will copy the files based on a list of file names in a target text file and place the copies in a file folder at the identified location. This script will not look at subdirectories in the target directory. To get the files of interest into a single file folder, see recursive_copy(). When using copy_by_list(), the user can either choose to submit the file path and target file list as arguments when initiating the function or can run the function with no arguments and be prompted for the necessary information. If running without arguments, the user will first be prompted to choose a file folder where the files of interest are located. Then it is necessary to select the target file with the list of desired files. The output file folder name will follow the format YYYYMMDD_copy_by_list, where the first eight characters represent the date of running, and the second section is in reference to the function name. The text file with the list of target files which the user wants to be copied into a single folder needs to have one file name per line and a single blank line at the end of the list.

### Sequence Manipulation

The manipulation of sequence data can also be a challenge when uploading to databases. This is especially true when dealing with large data sets containing multiple markers from different sources, researchers, or naming conventions. The following five R functions will help to manipulate multiple sequence fasta files. One note is that degap(), rank_seq(), head_derep(), and seq_derep() require a single line (not multiline) fasta input file for proper functioning. If the working file is in multiline format, the user can use multi_to_single_fasta() to convert it to single line format.

*degap()*

It is often desired to only upload unaligned data to public databases. To accomplish this easily we present the degap() tool. This function is designed to remove gaps (represented by "-") from all sequences in a selected fasta file. Users will need to select a file folder as a location to save the output file and an input fasta file. The user can either choose to submit the file path and the file they want to work on as arguments when initiating the tool or they can run the tool with no arguments and be prompted for the necessary information. The output file will follow the naming convention YYYYMMDD_degap.fas and be saved in the selected working directory.

*rank_seq()*

Often it is useful to screen out sequences of shorter length from further analyses. rank_seq() will take a multiple sequence fasta file and organize the sequences from shortest to longest. This will ease the visualisation of the fasta file in an alignment program and facilitate the selection of sequences over a given length and removal of sequences below a target length. This tool takes a select multiple sequence fasta file, organizes the sequences from shortest to longest, and saves the output in a new fasta file. Users will need to select a file folder as a location to save the output file and an input fasta file. The user can either choose to submit the file path and the file they want to work on as arguments when initiating the tool or, alternatively, can run the tool with no arguments and be prompted for the necessary information. The new sorted file will be saved in the selected location with the naming convention YYYYMMDD_rank_seq.fas.

*head_derep()*

Removing duplicate records based on the fasta file header may be necessary to ensure no repetition of data. head_derep() addresses this need. This function will reduce a select fasta file to all unique entries based on the headers. Users will need to select a file folder as a location to save the output file and an input fasta file. The user can either choose to submit the file path and the file they want to work on as arguments when initiating the tool or, alternatively, can run the tool with no arguments and be prompted for the necessary information. The output will be saved in the selected directory with the naming convention of YYYYMMDD_head_derep.fas.

*seq_derep()*

Removing duplicate records based on sequence may be necessary to ensure no repetition of data or when looking to determine the haplotype diversity in a multiple sequence file. This tool will reduce a select fasta file to all unique entries based on the sequences. Users will need to select a file folder as a location to save the output file and an input fasta file. The user can either choose to submit the file path and the file they want to work on as arguments when initiating the tool or, alternatively, can run the tool with no arguments and be prompted for the necessary information. The output will be saved in the selected directory with the naming convention of YYYYMMDD_seq_derep.fas.

*multi_to_single_fasta()*

Often, multiline fasta files where the header is on the first line followed by one or more lines of up to 80 characters containing nucleotide sequence data can be problematic when using different programs or tools. multi_to_single_fasta() can be used to change a multiple line fasta file format to a single line format where each header has a single line of nucleotide sequence data associated with a header. This tool will accept a multi-line fasta file and convert it to a single line fasta file format. Users will need to select a file folder as a location to save the output file and an input fasta file. The user can either choose to submit the file path and the file they want to work on as arguments when initiating the tool or, alternatively, can run the tool with no arguments and be prompted for the necessary information. The output will be saved in the selected directory with the naming convention of YYYYMMDD_multi_to_single_fasta.fas.

## Conclusion

This work is intended to assist scientists, technicians, and data managers to organize DNA barcode data and associated metadata for upload to public databases. A fundamental element of DNA barcoding is the presence of a centralized repository with diverse data providing an understanding of the within-species compared to between-species variation present in sequences. Since this information is essential to all barcode projects, a greater effort to populate these databases and make records public is necessary. Although this package and these nine functions are not comprehensive, they do represent a step forward in helping researchers move data to shared databases. This is especially true in organizations with secured networks where access to file manipulation via terminal windows is not possible. As such, we present the Molecular Data Organization for Publication (MDOP) R package to remove obstacles to these uploads.
